# Effect of baby food marketing exposure on infant and young child feeding regimes in Bangkok, Thailand

**DOI:** 10.1186/s13006-022-00503-7

**Published:** 2022-09-01

**Authors:** Nisachol Cetthakrikul, Matthew Kelly, Phillip Baker, Cathy Banwell, Julie Smith

**Affiliations:** 1 National Centre for Epidemiology and Population Health, Australian National University, Canberra, ACT Australia; 2 International Health Policy Program, Ministry of Public Health, Nonthaburi, Thailand; 3 Institute for Physical Activity and Nutrition, Deakin University, Geelong, Australia

**Keywords:** Baby food marketing, Infant and young child feeding behaviour, Thailand

## Abstract

**Background:**

Baby food marketing undermines breastfeeding by influencing women’s attitudes and decision-making favourably toward commercial baby food. This study aimed to explore the effects of various baby food marketing techniques on Thai mothers’ opinions about commercial milk formulas (CMF) and commercial complementary foods (CCF) and their infant and young child feeding behaviours.

**Methods:**

This study used a cross-sectional survey employing the World Health Organization (WHO) NetCode Toolkit Protocol for Periodic Assessment, and the United Nations International Children’s Emergency Fund (UNICEF) Multiple Indicator Cluster Survey to collect data on mothers’ experience with and their opinion on the various types of marketing of CMF and CCF, and their feeding behaviour. Data collection used structured interviews of mothers with children aged two years or below attending 33 health facilities in Bangkok. Univariable and multivariable regression analysis then investigated links between mothers’ reported exposure to baby food marketing and their infant and young child feeding behaviours, employing a semantic scale and considering key sociodemographic and other variables.

**Results:**

Three hundred and thirty mothers were surveyed in Bangkok. Around 90% reported experiencing exposure to at least one type of baby food marketing during the previous six months, mostly from electronic media. More than half of the women had positive opinions of CMF. Virtually all children had been breastfed initially, but 74.6% were given CMF and 72.8% stopped breastfeeding before six months. Multivariable analysis showed that mothers who lived in a couple were significantly less likely to favour CMF, and mothers in middle-income households and those who had received advice about CMF from others were more likely to have a favourable opinion. Mothers in formal employment were over six times more likely to feed formula than those not in employment. Women who experienced baby food marketing at health facilities were four times more likely to feed CMF to their children than those not experiencing such marketing.

**Conclusions:**

Specific types of baby food marketing were strongly linked to mothers’ opinions on and use of CMF in Bangkok, Thailand. It is recommended that breastfeeding policies in health facilities and employment are fully implemented and enforced.

**Supplementary Information:**

The online version contains supplementary material available at 10.1186/s13006-022-00503-7.

## Background

In 2019, the Thailand Multiple Indicators Cluster Survey found that only 34% of mothers and babies initiated breastfeeding within one hour of birth. The exclusive breastfeeding rate of under-six-month-old infants was 14%. The proportions who were breastfed until one year and two years old, were 24% and 15%, respectively. These breastfeeding rates in Thailand were far lower than the 2030 Global Target for breastfeeding [1]; breastfeeding initiation of 70%; exclusive breastfeeding of 70%; and continued breastfeeding until one year and two years of age of 80% and 60% respectively [2].

Thailand has policies to protect, promote, and support breastfeeding. For instance, the Labour Protection Act B.E. 2541 states that working mothers are entitled to 98 days of maternity leave with full pay [3]. The Baby-Friendly Hospital Initiative (BFHI) has been implemented since 1992 in health facilities, particularly public hospitals, to deliver maternal-and-child health services following the “Ten Steps to Successful Breastfeeding” [3, 4]. Furthermore, Thailand adopted and implemented The International Code of Marketing of Breastmilk Substitutes (the Code) as a voluntary measure in 1981. However, there were no penalties for non-compliance by companies.

In 2010, the World Health Assembly (WHA) resolution 63.23 urged all member states to fully adopt the Code into national law [5]. A further resolution WHA 63.14 called for action to minimise the impact of unhealthy food marketing on children, by restricting such marketing, including in settings where children gather such as schools, without conflicts of interest [6]. Therefore, Thailand implemented the Control of Marketing Promotion of Infant and Young Child Food Act B.E. 2560 (the Act) in 2017. The Act prohibits the promotion of baby food such as advertising, cross-promotion, and direct contact with mothers. Furthermore, the Act has restrictions on baby food marketing in the health system, for example, through donations, sponsorship, and offering medical equipment. Most provisions of the Act follow the Code, but due to the interference from baby food industries, for example, lobbying, building relationships with policymakers, and seeking involvement in working groups, technical groups and advisory groups during the legislation process of the Act, some provisions of the Act are different from the Code, such as the scope of products. The Act does not include growing-up milks (GUM) and bottles and teats [7].

Since that time, the new Act implemented the Code in law in Thailand, although it has been illustrated as noted above that corporate political activities of baby food companies influenced the legislation process of the Act [7]. It has also been demonstrated that the companies did not comply with Thai law or the Code so that exposure of the public and new mothers to marketing of baby food products including GUM remains very high [8].

The World Health Organization (WHO) defines marketing as “any form of commercial communication or message that is designed to, or has the effect of, increasing recognition, appeal and / or consumption of particular products and services” [9]. Marketing activity includes various forms of promotion such as advertising and is conducted through different channels, such as via health facilities or electronic media. In 2016, technical guidance issued by the WHO confirmed that the definition of BMS included in the Code was “... any milks (or products that could be used to replace milk, such as fortified soy milk), in either liquid or powdered form, that are specifically marketed for feeding infants and young children up to the age of 3 years (including follow-up formula and growing-up milk)” [10]. As well, the guidance defined that promotion of food for infants and young children is inappropriate, if it interferes with breastfeeding [10].

As breastfeeding indicators improved in high-income countries [11] and regulations to restrict the marketing of BMS were strengthened, baby food marketing has increasingly focused on middle-income countries in Asia. There was a significant increase in global commercial milk formula (CMF) sales from 3.5 to 7.4 kg per child between 2005–19 [12]. A recent study [13] in upper-middle-income countries found that between 2000–19, there was a significant increase of 0.56 percentage points in the absolute average annual changes of formula consumption among infants up to six months. There was high consumption of CMF and CCF in Asian countries, for example, the percentage of prevalence of prelacteal feeding at discharge after delivery in Kathmandu Valley, Nepal was 55.9% in 2014 [14]. Second, in 2014, around 43% of Cambodian mothers of 0–5-month old infants reported they provided breastmilk substitutes to their child [15].

In such countries as Thailand market expansion possibilities are greater than in high income countries [16]. In Thailand, Euromonitor sales data indicates that households are increasingly purchasing milk formula. Between 2015–20 the percentage of volume growth of infant formula (for ages 0–6 months), follow-on formula (7–12 months), and growing-up formula (13–36 months) was 4, 5.3, and 11.3, respectively. Formula retail sales rose from 24,615.1 million Baht (USD$724.91million) in 2013, to 31,712.5 THB million (US$933.93million) in 2020 [17, 18]. Meanwhile, the percentage of exclusive breastfeeding in Thailand decreased from 23% in 2016 [19] to 14% in 2019 [1].

Inappropriate baby food marketing affects the feeding behavior of mothers because such marketing can positively change social norms and caregivers’ attitudes toward formula feeding. For example, A recent study in Indonesia showed that a high prevalence of marketing including through health systems was associated with perceived milk insufficiency, and mistaken maternal motivations for feeding BMS such as growth, intelligence and immunity. It was also associated with maternal employment outside the home. Such research findings highlight that as well as information and counselling, and marketing, broader social health system and employment environments are important for whether mothers’ intentions can be translated into infant feeding practice [20].

Furthermore, Social Cognitive Theory identifies the key factors relating to behaviour change. One is individual factors such as knowledge, and self-efficacy. Another is the existence of supportive factors or barriers in the environment such as family and community, and health system services [21, 22]. Likewise, the Theory of Planned Behaviour presents that infant feeding behaviour relates not only to attitude, intention and subjective norms but also to perceived behavioural control [21]. That is, unless the broader social and home environments support breastfeeding, women may not be enabled to give effect to their breastfeeding intentions.

Hence, apart from baby food marketing, maternal factors such as intention, knowledge, experience in, confidence, and self-efficacy of breastfeeding, also have sociodemographic associations with infant feeding practice. Previous studies in Asia and Africa showed that strong intention to breastfeed [23], and receiving breastfeeding information via counselling during pregnancy [24] were enabling factors of breastfeeding. However, those with low or no education [25, 26], or high income mothers who could afford to buy commercial baby foods rarely maintain exclusive breastfeeding [26].

Until recently little research has directly addressed the associations between baby food marketing and mothers’ infant feeding attitudes and behaviours, allowing companies to dispute their influence on breastfeeding despite enormous marketing expenditures on baby food marketing and promotion targeting mothers, and their families. This denial is a tactic employed by other industries such as the tobacco industry [27]. In 2015, Piwoz et.al presented a broad conceptual framework for the effect of BMS marketing on breastfeeding practices. This framework traced how baby food marketing links to positive attitudes toward baby food marketing and sub-optimal breastfeeding practice [28].

However, there is now growing evidence including from cross-sectional studies showing empirical data linking baby food marketing directly with behavior change. For example, in the Philippines, mothers who recalled formula advertising messages were shown to be more likely to give formula to their children than those who did not recall such messages [29]. Likewise, in the USA, mothers exposed to infant formula information from the media both offline and online were more likely to intend to use infant formula or use formula earlier compared with mothers who did not receive formula information [30]. Moreover, a 2015 study in Thailand found that mothers who more frequently perceived marketing of CMF, were more likely to have positive attitudes toward such marketing, and these mothers were more likely to feed CMF to their children [31].

There are multiple techniques used to market baby food. These include, for example, direct or indirect contact with pregnant or lactating women, digital marketing, and product packaging and labelling. Interestingly, many countries control marketing of infant and follow-on formula [32], but allow growing-up milk to be promoted. Therefore, baby food marketing increasingly uses cross-promotion techniques by promoting growing-up milk to link to infant or follow-on formula. Consequently, caregivers may confuse infant formula and growing-up milk. A study in Australia found that sampled women were not able to distinguish between advertising for infant formula and for GUM [33] There is strong recent evidence from Indonesia [34] and the US [34] showing that GUM contains sugar at levels of serious concern, and is unsuitable for inclusion in the diets of young children. Exposure to marketing claims increased parents’ intentions to give the product, increased its perceived healthfulness, and resulted in parents’ wrong perceptions that it had medical endorsement. Similarly in Vietnam, exposure to marketing of unhealthy commercial milk formula for pregnant women (CMF-PW), creating beliefs that such products were widely used and would make a child smart and healthy, were shown to be associated with greater use of such products [35].

This study, therefore, aimed to explore the associations between exposure to various types of baby food marketing on mothers’ opinions on formula and practices regarding formula feeding in Bangkok, Thailand three years after the new Thai law, in 2020.

It focused on mothers who live in Krung Thep Maha Nakhon (Bangkok), the capital city of Thailand, because baby food companies have more possibility to market their products in the capital city or big cities than other regions of countries [36]. Therefore, mothers in Bangkok may be more likely to have experienced baby food marketing. Moreover, the percentages of breastfeeding initiation and continue breastfeeding for two years of age in Bangkok were around 21%, and 4% respectively [1] which were lower than for other regions in Thailand.

## Methods

This cross-sectional quantitative study employed the WHO NetCode Toolkit Protocol for Periodic Assessment [36] to design data collection on the marketing of baby food to mothers in Bangkok. The NetCode Toolkit Protocol for Periodic Assessment is a part of a toolkit developed by the WHO to assist governments in establishing a sustainable system that will monitor, detect and report violations of national laws and the International Code of Marketing of Breastmilk Substitutes [36].

In regard to feeding history, we adapted questions from the Thailand Multiple Indicator Cluster Survey (MICS) which is a widely used household survey developed by the United Nations International Children’s Emergency Fund (UNICEF) to support countries in data collection about the situation of children and women [1].

We employed a semantic scale to explore mothers’ attitudes toward breastmilk substitute marketing more fully, and to lessen social desirability response bias. A semantic scale uses words rather than numbers to describe respondents’ attitudes such as toward products [37]. In this study, we measured mothers’ attitudes to CMF, and each type of baby food marketing. The left polar of scale represented ‘unfavourable’, while the right polar of scale showed ‘favourable’.

### Sample selection

Mothers of children aged less than or equal to 24 months were the unit of analysis for this study and were recruited via health facilities according to the Netcode Protocol. This study selected health facilities from the same sample group as a previous study ‘Marketing of Breast-Milk Substitutes Thailand’, conducted by the Access to Nutrition Foundation, Westat, and the International Health Policy Program in 2017 [38]. This previous study in 2017 also followed the NetCode Toolkit Protocol for Periodic Assessment [36] to select health facilities. A two-stage sample design was employed to select health facilities [39].

Our study selected 33 main and 12 back-up health facilities from the previous study [39], and the first author contacted all of the main health facilities to ask for permission to conduct data collection. If the main health facilities did not allow the collection of data, back-up health facilities, which did allow the collection of data, were used instead. Finally, 31 public health facilities and 2 private hospitals, located in 25 out of the 50 districts across Bangkok, were included in this study.

In each health facility, all mothers with a 0–2-year-old child at well-baby clinics (vaccination clinics) were asked for their formal consent, and the first ten mothers who agreed to participate were included.

Well-baby clinics are a unit of health facilities that provide health promotion services for healthy infants and children including vaccination, growth monitoring and development. Therefore, parents take their children to the well-baby clinics to follow the vaccination schedule for young children (0–12 years old) [40]. Our targeted samples were mothers of two-year-old or under children; thus, they had to visit the well-baby clinics.

### Survey tools

This study employed structured face-to-face electronic questionnaires to collect data from mothers. The questionnaire was developed using a NetCode Toolkit Protocol [36] to collect information on mothers and their experience in BMS marketing. Moreover, we separately collected feeding history of breastfeeding, formula feeding and complementary feeding using selected infant feeding questions from the Thailand MICS. A semantic scale [37] was applied to record the attitudes of mothers toward baby food marketing which they have experienced, and attitudes to CMF.

The baby food marketing exposures included in this study were: advice about formula from others; advice about commercial complementary food (CCF) from others; marketing from health facilities; marketing from media; companies' social group and events; free baby food samples; free coupons relating to baby food products or companies; and free gifts relating to baby food products or companies.

All data were collected and recorded on a tablet through Kobocollect [41], which is a data collection application.

### Data collection

The lead researcher recruited bachelor’s or master’s degree students who then attended a three-day training course to become data collectors for the study. They learned about the Code, the Act, baby food, baby food marketing, and baby food companies in Thailand. Furthermore, they learned about the questionnaire format and the data collection process, and they engaged in role-playing in a pilot set to ensure that they were able to collect data correctly.

Data collection was conducted between 3 and 19 March 2020 and between 18 June and 4 August 2020. The COVID-19 pandemic movement restrictions caused fieldwork to stop for two months. The data collection team consisted of one field manager, a key researcher with experience in similar data collection, and three data collectors.

Selected health facilities were contacted to make an appointment for data collection at well-baby clinics. At the waiting room of the well-baby clinics that all mothers and children employ while waiting for vaccination service, the data collection team approached asked mothers, to select whose child was aged less than or equal to 24-months old, and then asked for their consent to participate in this study.

A total of 330 participating mothers from selected health facilities were then asked for information such as their age, education level, marital status, number of household members, household income, and occupation. Such factors are known to influence infant feeding decisions by affecting mothers’ knowledge and attitudes, as well as the infant feeding decisions that are practically open to them.

Also, researchers asked mothers about where they gave birth, and about their experiences in baby food marketing, feeding history (starting point and ending point of feeding breastmilk, formula, home-prepared complementary food, and commercially-prepared complementary food), and what they feel about formula and baby food marketing, to explore their attitudes to formula and baby food marketing. These factors relate to facility policies and practices which influence mothers’ likelihood of exposure to marketing at the critical time for establishing exclusive breastfeeding, as well as individual attitudinal factors affecting decisions about using formula.

### Data analysis

The analysis was divided into three steps: descriptive statistics, univariable analysis, and multivariable analysis.

Descriptive statistics were used to analyse the sociodemographic characteristics of mothers, mothers’ experience of baby food marketing, mothers’ opinions on baby food marketing, and their feeding behaviour.

Univariable analysis was used to determine the association between mothers’ opinions on formula milk and their formula feeding behaviour and these sociodemographic and marketing exposure variables. The independent variable in these steps were mothers’ characteristics, namely age, education level, marital status, the number of family members, type of household, employment, income, and child’s place of birth. Variables addressing the type of marketing experienced included: advice about formula or commercially-prepared complementary food; marketing at health facilities; digital marketing; the maternal group hosted by BMS companies; free samples; free coupons; and gifts; which were also included as independent variables.

Similarly, multivariable analysis was used to measure associations between baby food marketing and mothers’ opinion on formula and relationships between baby food marketing and formula feeding behaviour after mutually adjusting for the effect of all variables at the same time. All variables from the univariable analysis were added to the multivariable analysis, even though they did not reveal statistically significant associations in terms of adjusted odds ratios. This is because previous studies showed that multiple maternal factors such as education level or age or poverty and baby food marketing are related to breastfeeding practice [28, 42, 43].

All analyses were performed using STATA (version 14.2).

## Results

### Characteristics of mothers

Most mothers were aged 20–29 years, and their education level was secondary school and Diploma level. Furthermore, most mothers were married or lived with their partners, and about half of them were not in paid employment. In terms of households, about half lived in extended families. Most lived in families with no more than five members, and the amount of household monthly income was mainly between 15,001–50,000 THB (USD 480.1–1,600.2). Regarding child characteristics, most children were aged six months or more, and the majority were born at public health facilities (Table 1).

**Table 1:** Characteristics of mothers

Characteristics of mothers	N (%)
**Children’s age (*n* = 330)**	
Child < 6 months	98 (29.7)
Child > = 6 months	232 (70.3)
**Mothers’ age (*n* = 330)**	
< 20 years	31 (9.4)
20–29 years	179 (54.2)
30 years and above	120 (36.4)
**Education level (*n* = 327)**	
Primary school or lower	55 (16.8)
Secondary school or diploma	224 (68.5)
Bachelor’s degree or higher	48 (14.7)
**Marital status (*n* = 330)**	
Live without couple	30 (9.1)
Live as a couple	300 (90.9)
**Total number of household members (*n* = 330)**	
1–5 persons	224 (67.9)
6 persons and above	106 (32.1)
**Type of household (*n* = 329)**	
Nuclear family	164 (49.8)
Extended family	165 (50.2)
**Current occupation / employment status of mothers (*n* = 327)**	
Non-employed / student	169 (51.7)
Formal work	89 (27.2)
Informal work	69 (21.1)
**Household monthly incomes (*n* = 330)**	
0–15,000 THB	95 (28.8)
15,001–50,000 THB	190 (57.6)
More than 50,000 THB	45 (13.6)
**Type of hospital where children were born (*n* = 326)**	
Public hospitals / clinics	278 (85.3)
Private hospitals / clinics	48 (14.7)

### Mothers’ experience of others’ advice or marketing of baby food

Just under 90% of mothers (*n* = 296) reported that they had experienced at least one type of baby food marketing or received others’ advice relating to baby food. Mostly this was through the media (82.2%), but it was commonly also by joining maternal groups or events, where 30.5% had received promotions. The third-highest percentage of mothers who reported experiencing baby food marketing (26.1%) were those who received a free baby food sample (Table 2). In terms of receiving advice from others, 22.5% and 20.6% of mothers were provided advice about formula or complementary food for infants or children from 6–36 months sold at retail outlets, from others — for example, health professionals, families, or friends (Table 2).

**Table 2:** Mothers’ experiences of baby food advice and marketing, by type of exposure

Types of marketing experienced	N (%)
**Mothers’ experiences of others’ advice or marketing of baby food (*n* = 330)**	
Advice about formula from others	74 (22.5)
Advice about commercially-prepared complementary food from others	68 (20.6)
Marketing from health facilities	74 (22.7)
Marketing from electronic media	267 (82.6)
Companies’ social groups and events	39 (30.5)
Free baby food sample	86 (26.1)
Free coupons relating to baby food products or companies	24 (7.3)
Free gifts relating to baby food products or companies	39 (11.9)
**Opinion of mothers on others’ advice or marketing of baby food**	
Advice about formula from others **(*n* = 74)**	
Extremely unfavourable	0 (0.00)
Quite unfavourable	1 (1.3)
Slightly unfavourable	4 (5.4)
Neither	30 (40.5)
Slightly favourable	11 (14.9)
Quite favourable	15 (20.3)
Extremely favourable	13 (17.6)
Advice about commercially-prepared complementary food from others **(*n* = 68)**	
Extremely unfavourable	4 (5.9)
Quite unfavourable	4 (5.9)
Slightly unfavourable	2 (2.9)
Neither	27 (39.7)
Slightly favourable	11 (16.2)
Quite favourable	14 (20.6)
Extremely favourable	6 (8.8)
Marketing from health facilities **(*n* = 74)**	
Extremely unfavourable	0 (0.00)
Quite unfavourable	1 (1.3)
Slightly unfavourable	0 (0.00)
Neither	29 (39.2)
Slightly favourable	17 (23.0)
Quite favourable	12 (16.2)
Extremely favourable	15 (20.3)
Marketing from the media **(*n* = 267)**	
Extremely unfavourable	1 (0.4)
Quite unfavourable	2 (0.7)
Slightly unfavourable	4 (1.5)
Neither	137 (51.3)
Slightly favourable	39 (14.6)
Quite favourable	57 (21.4)
Extremely favourable	27 (10.1)
Being a member of online or in-person mothers’ group sponsored by baby food companies **(*n* = 37)**	
Extremely unfavourable	0 (0.00)
Quite unfavourable	0 (0.00)
Slightly unfavourable	1 (2.7)
Neither	10 (27.1)
Slightly favourable	7 (18.9)
Quite favourable	12 (32.4)
Extremely favourable	7 (18.9)
Marketing from online or in-person mothers’ group **(*n* = 29)**	
Extremely unfavourable	0 (0.00)
Quite unfavourable	0 (0.00)
Slightly unfavourable	0 (0.00)
Neither	11 (37.9)
Slightly favourable	4 (13.8)
Quite favourable	8 (27.6)
Extremely favourable	6 (20.7)
Participation in events or activities hosted for mothers sponsored by baby food companies **(*n* = 14)**	
Extremely unfavourable	0 (0.00)
Quite unfavourable	0 (0.00)
Slightly unfavourable	1 (7.2)
Neither	3 (21.4)
Slightly favourable	1 (7.2)
Quite favourable	3 (21.4)
Extremely favourable	6 (42.8)
Marketing from events or activities hosted for mothers **(*n* = 13)**	
Extremely unfavourable	0 (0.00)
Quite unfavourable	0 (0.00)
Slightly unfavourable	0 (0.00)
Neither	6 (46.1)
Slightly favourable	1 (7.7)
Quite favourable	2 (15.4)
Extremely favourable	4 (30.8)
Free baby food sample **(*n* = 86)**	
Extremely unfavourable	2 (2.3)
Quite unfavourable	0 (0.00)
Slightly unfavourable	1 (1.2)
Neither	30 (34.9)
Slightly favourable	12 (14)
Quite favourable	23 (26.7)
Extremely favourable	18 (20.9)
Free coupons relating to baby food products or companies **(*n* = 24)**	
Extremely unfavourable	1 (4.1)
Quite unfavourable	0 (0.00)
Slightly unfavourable	0 (0.00)
Neither	10 (41.7)
Slightly favourable	4 (16.7)
Quite favourable	4 (16.7)
Extremely favourable	5 (20.8)
Free gifts relating to baby food products or companies **(*n* = 39)**	
Extremely unfavourable	1 (2.6)
Quite unfavourable	0 (0.00)
Slightly unfavourable	0 (0.00)
Neither	8 (20.5)
Slightly favourable	5 (12.8)
Quite favourable	18 (46.2)
Extremely favourable	7 (17.9)

### Opinions of mothers on others’ advice or marketing of baby food

Mostly, mothers had a neutral attitude toward receiving advice about formula and commercially-prepared complementary food from others, and also to almost all types of baby food marketing. The exception was that most mothers (32.4%) had quite positive opinions on being a member of mothers’ groups. Likewise, 42.8% of mothers had extreme positive opinions on participating in events sponsored by a baby food company. Moreover, most mothers (46.2%) quite liked receiving gifts relating to baby food products. (Table 2).

### Influences on mothers’ opinion on formula of others’ advice or marketing of baby food

More than half of mothers had positive opinions about formula, with the exceptions more likely to be mothers who had a bachelor’s degree or higher (47.9%); mothers who lived in large households (48.1%); or mothers who had low or high monthly incomes (49.5% and 33.3%, respectively) (Table S1). More than 50% of mothers had positive opinions on formula if they had experienced baby food marketing such as receiving advice from other people relating to formula or complementary food or joining maternal groups or events, or receiving free samples or coupons or gifts (Table S1).

In the univariable analysis, mothers in large households (with six or more members) had a significantly lower likelihood of being positive about formula compared with mothers in smaller households (OR = 0.61). In contrast, mothers in the middle-income group (household income between 15,001 and 50,000 THB) had an odds ratio of 1.92 (95% CI 1.16, 3.17; *p* = 0.01) compared to mothers with a lower, or higher household income (less than 15,000 THB or more than 50,000 THB). Likewise, mothers who had received advice about formula had a higher likelihood of holding positive attitudes about formula than mothers who had not received advice about formula (Table 3).

**Table 3:** Regression analyses of associations between sociodemographic characteristics and baby food marketing experience, and mothers’ positive opinions toward formula feeding

**Factors**	**Positive opinion of mothers on** **formula**					
	**Univariable analysis**			**Multivariable analysis**		
	**OR**	**95% CI**	***P*-value**	***A*OR**	**95% CI**	***P*-value**
*Sociodemographic characteristics and opinions on formula*						
**Mothers’ age (v < 20 years)**						
20–29 years	1.09	0.51, 2.35	0.82	1.23	0.35, 4.33	0.74
30 years and above	1.04	0.47, 2.30	0.92	0.6	0.13, 2.69	0.5
**Education level (v primary school or lower)**						
Secondary school or diploma	1.07	0.59, 1.94	0.82	0.2	0.03, 1.36	0.1
Bachelor’s degree or higher	0.71	0.33, 1.55	0.39	0.21	0.02, 2.09	0.18
**Marital status (v live without couple)**						
Live as a couple	0.99	0.46, 2.10	0.97	0.22	0.05, 0.94	0.04
**Total number of household members (v 1–5 persons)**						
6 persons and above	0.61	0.38, 0.97	0.04	0.4	0.14, 1.12	0.08
**Type of household (v nuclear family)**						
Extended family	0.87	0.56, 1.35	0.54	0.74	0.25, 2.22	0.59
**Current occupation / employment status of mothers (v non-employed / student)**						
Formal work	0.97	0.58, 1.64	0.92	0.75	0.29, 1.95	0.55
Informal work	0.99	0.56, 1.74	0.97	1.12	0.34, 3.71	0.86
**Household monthly incomes (v 0–15,000 THB)**						
15,001–50,000 THB	1.92	1.16, 3.17	0.01	7.92	2.39, 26.21	0.00
More than 50,000 THB	0.51	0.24, 1.07	0.08	3.37	0.78, 14.45	0.10
**Types of a hospital where children were born (v public hospitals / clinics)**						
Private hospitals / clinics	0.91	0.49, 1.69	0.77	1.95	0.64, 5.94	0.24
***Experience in marketing and opinions on formula***						
**Advice about formula from others (v no experience)**						
Having experience	1.82	1.06, 3.14	0.03	2.69	1.06, 6.80	0.04
**Advice about commercially-prepared complementary food from others (v no experience)**						
Having experience	1.05	0.61, 1.80	0.85	0.46	0.17, 1.27	0.13
**Marketing from health facilities (v no experience)**						
Having experience	0.78	0.46, 1.31	0.35	1.11	0.43, 2.89	0.83
**Marketing from media (v no experience)**						
Having experience	0.92	0.52, 1.63	0.77	1.33	0.33, 5.32	0.69
**Companies' social group and events (v no experience)**						
Having experience	1.11	0.56, 2.16	0.77	1.24	0.52, 2.95	0.63
**Free baby food sample (v no experience)**						
Having experience	1.1	0.67, 1.81	0.7	0.82	0.33, 2.03	0.67
**Free coupons relating to baby food products or companies (v no experience)**						
Having experience	1.31	0.55, 3.08	0.54	1.06	0.28, 4.04	0.94
**Free gifts relating to baby food products or companies (v no experience)**						
Having experience	1.25	0.63, 2.49	0.52	0.97	0.30, 3.15	0.96

*AOR* Adjusted odds ratio, *OR* Odds ratio, *v* Versus

Findings of the multi-regression analysis illustrated that mothers who lived with a partner or husband were significantly less likely to have a positive opinion on formula than mothers who did not live as a couple (*p* = 0.04). In contrast, mothers reporting household income between 15,001 and 50,000 THB (*p* = 0.00), or mothers who had received advice about formula had a higher likelihood of having a positive opinion, with adjusted odds ratios of 7.92 (95% CI 2.39, 26.21; *p* = 0.00) and 2.69 (95% CI 1.06, 6.80; *p* = 0.04) respectively (Table 3).

### Feeding behaviours

Just over 97% of children had ever been breastfed. However, 43.8% of them had stopped breastfeeding at between two or three months of age (Table S2). Most children (74.5%) had been given formula, and approximately 33% of these children had started formula between two and three months of age, and around 29% of them started formula at 0–1 month. Moreover, 39.4% of children were introduced to commercially-prepared complementary food, whereas 68.2% of children were fed home-prepared complementary foods. Most of them were introduced to complementary food after six months, being commercially-prepared complementary food (64.6%), and home-prepared complementary food (68.5%) (Table S2).

### Influences on formula feeding of others’ advice or marketing of baby food

A high proportion of mothers who reported receiving advice relating to formula (73%) or complementary food (83.8%) from other people introduced formula to their child. In the same way, most mothers who experienced baby food marketing such as marketing in health facilities, digital marketing, and who received free samples, or coupons, fed formula to their child (Table S3).

Findings from univariable regression analysis illustrated that mothers who experienced baby food marketing from the media were around twice as likely to feed formula to their child than mothers who reported they had never seen baby food marketing from the media (95% CI 1.43, 4.73; *p* = 0.00). Mothers working in the formal sector were approximately three times more likely to feed formula to their children than mothers who were not employed (95% CI 1.50, 6.24; *p* = 0.00). Informal employment did not affect the likelihood of feeding formula (Table 4). The level of families’ incomes was associated in a complex way with the likelihood of formula feeding. Mothers in a family earning 15,001–50,000 THB per month had a higher likelihood of feeding their child with formula than mothers who had lower household incomes of 15,000 THB or below (95% CI 1.01, 2.99; *p* = 0.05), or compared to very high-income households. Furthermore, children born at private health facilities were twice as likely to be fed formula than children born at public health facilities (95% CI 1.06, 6.33; *p* = 0.04) (Table 4).

**Table 4:** Regression analyses of associations between sociodemographic factors and experience of baby food marketing, and giving formula

**Factors**	**Giving** **formula**					
	**Univariable analysis**			**Multivariable analysis**		
	**OR**	**95% CI**	***P*-value**	***A*OR**	**95% CI**	***P*-value**
*Sociodemographic factors, and giving formula*						
**Mothers’ age (v < 20 years)**						
20–29 years	0.71	0.28, 1.85	0.49	0.82	0.18, 3.73	0.80
30 years and above	0.63	0.24, 1.68	0.36	0.39	0.06, 2.41	0.31
**Education level (v primary school or lower)**						
Secondary school or diploma	1.34	0.70, 2.56	0.37	0.81	0.12, 5.47	0.83
Bachelor’s degree or higher	1.94	0.77, 4.88	0.16	2.24	0.13, 40.13	0.58
**Marital status (v live without couple)**						
Live as a couple	0.56	0.21, 1.51	0.25	1.21	0.22, 6.74	0.83
**Total number of household members (v 1**–**5 persons)**						
6 persons and above	0.93	0.55, 1.57	0.78	1.06	0.33, 3.44	0.92
**Type of household (v nuclear family)**						
Extended family	1.26	0.77, 2.08	0.36	0.91	0.25, 3.33	0.89
**Current occupation / employment status of mothers (v non-employed / student)**						
Formal work	3.06	1.50, 6.24	0.00	6.37	1.49, 27.31	0.01
Informal work	0.99	0.54, 1.82	0.97	1.63	0.42, 6.35	0.48
**Household monthly incomes (v 0–15,000 THB)**						
15,001–50,000 THB	1.74	1.01, 2.99	0.05	1.94	0.53, 7.03	0.32
More than 50,000 THB	2.03	0.87, 4.73	0.10	0.82	0.16, 4.20	0.81
**Types of a hospital where children were born (v public hospitals / clinics)**						
Private hospitals / clinics	2.59	1.06, 6.33	0.04	1.25	0.29, 5.41	0.76
*Experience in baby food advice and marketing, and giving formula*						
**Advice about formula from others (v no experience)**						
Having experience	0.90	0.50, 1.63	0.74	0.99	0.33, 2.96	0.99
**Advice about commercially-prepared complementary food from others (v no experience)**						
Having experience	2.00	0.99, 4.03	0.05	0.86	0.25, 3.03	0.82
**Marketing from health facilities (v no experience)**						
Having experience	1.45	0.77, 2.73	0.25	4.44	1.15, 17.09	0.03
**Marketing from media (v no experience)**						
Having experience	2.60	1.43, 4.73	0.00	2.59	0.62, 10.78	0.19
**Companies' social group and events (v no experience)**						
Having experience	0.64	0.28, 1.45	0.28	0.62	0.21, 1.85	0.39
**Free baby food sample (v no experience)**						
Having experience	1.28	0.71, 2.29	0.41	0.67	0.23, 1.93	0.46
**Free coupons relating to baby food products or companies (v no experience)**						
Having experience	2.49	0.72, 8.57	0.15	2.1	0.36, 12.39	0.41
**Free gifts relating to baby food products or companies (v no experience)**						
Having experience	0.84	0.40, 1.78	0.66	0.77	0.17, 3.38	0.73

*AOR* Adjusted odds ratio, *OR* Odds ratio, *v* Versus

Multivariable analysis of the relationship between mothers’ reported experiences of marketing and their feeding behaviour is reported in Table 4. The results of this analysis revealed that mothers in formal work at the time of the interviews were around six times more likely (AOR = 6.37) to give formula than mothers who were not employed (95% CI 1.49, 27.31; *p* = 0.01). Similarly, mothers who had received baby food marketing at health facilities had around a four times higher likelihood of formula feeding than mothers who had not seen baby food marketing at health facilities (95% CI 1.15, 17.09; *p* = 0.03) (Table 4).

## Discussion

This study found that marketing exposure of mothers was associated with favourable opinions about commercial baby food products. More than half of the mothers surveyed had favourable opinions on CMF and those with greater exposure to such promotional activity had much more favourable opinions and behaviours toward formula. Mothers’ opinions were most receptive to baby food marketing experienced through baby clubs and events, and gifts, but experiences of marketing in health settings, and factors such as maternal employment significantly influenced the use of CMF.

High levels of exposure to marketing in Thailand via a variety of techniques including electronic media is perhaps not surprising. It is known that baby food companies in Thailand have previously employed such techniques to promote their products, such as cross-promotion via similar labelling of infant formula and GUM, and via electronic media advertising which builds on previous consumer engagement on online platforms such as Google, Facebook and online retail outlets [44]. Earlier research using the same NetCode Protocol in 2017 found high levels of non-compliance with the Code, which at that time was primarily point-of-sale promotions. Previous study findings suggest the particular, growing importance of electronic media advertising and promotion in exposing mothers to marketing in 2020 [45]. This aligns with increasing global trends and heightened international policy concerns about digital marketing of BMS [46].

Widespread exposure to baby food promotion can change social norms and attitudes toward formula feeding, and lead consumers to believe that formula feeding is common and a social norm [28]. The analysis by Piwoz and Huffman, suggests that baby food marketing is intended to widely influence societal attitudes about safety and benefits of infant formula, for example, presenting infant formula as equal to or better than breastmilk, or as solving a child’s health problems such as a digestive problem [28]. For example, nearly 80% of mothers in Lao PDR were exposed to Thailand media's promotion of formula milk through TV commercials, and this exposure developed mothers’ positive attitudes toward formula milk and negatively affected breastfeeding in this neighbouring country [47].

According to a previous study, one of the reasons for mothers buying formula was the influence of a family member [48]. Other recent studies also found that family members were key influences on breastfeeding, for example, good support from husbands or partners has positive effects on successful exclusive breastfeeding [24], and wrong feeding advice from family members had negative effects [49]. This study shows that mothers’ attitudes in Thailand are shaped by prevailing social norms favouring formula feeding. The analyses of associations between baby food marketing and mothers’ opinions in this study illustrated that mothers receiving advice about formula from family members tended to favour formula. Mothers’ partners, relatives, or friends were identified as the groups of people who mostly recommended formula to mothers.

Exposure to baby food marketing was significantly associated with higher rates of formula feeding in this study. Univariable analysis showed that baby food marketing through the media was positively correlated with giving formula. This aligns with a previous study in the Philippines which showed that mothers who recalled advertising messages from TV were twice as likely to use formula [50]. Health professionals’ advice was also important in influencing the introduction of formula in this Philippines study. As well, in a study in Nepal, mothers who received recommendations from health professionals about using breastmilk substitutes had more possibility to provide prelacteal feed to their child than mothers who did not receive such recommendations [14]. Similarly, in multivariable regression, Thai mothers who noticed baby food marketing in health facilities were four times more likely to feed formula to their children than those who were not. According to *The Lancet* breastfeeding series [51], health systems and services are a key determinant of breastfeeding since health professionals can importantly influence and support mothers for breastfeeding decisions before and after birth. Furthermore, socioeconomic factors are important influences on the use of CMF.

In this study in Thailand, mothers living in middle-income households were several times more likely to use formula. This is consistent with findings from a study in Nigeria [52] which revealed that mothers from wealthier households were more likely to engage in bottle-feeding than mothers from poorer households. A previous study in Bangladesh also found that improved economic status was one of the crucial factors relating to increased formula feeding. In other words, poor families may not be able to afford formula or the product is a symbol of higher socioeconomic status [53].

This study also found that mothers in formal employment had a higher probability of giving formula. *The Lancet* breastfeeding series highlighted that working mothers were likely to stop breastfeeding or start weaning early, and this related to the short duration of maternity leave [51]. In previous studies, mothers who return to work in the first six months are less likely to exclusively breastfeed as recommended [23, 54, 55]. A recent International Labour Organization study showed that while Thailand provides some important key maternity protections, including its paid maternity leave, it did not meet any important labour standards for protecting breastfeeding at work through nursing break entitlements [56].

Around the world, many countries implement measures intended to restrict baby food marketing to protect families and especially mothers from such commercial pressures that reduce breastfeeding [32]. It is also well recognised that the policies and practices in maternity care facilities importantly affect breastfeeding, including the extent to which marketing is permitted and the use of breastmilk substitutes is accepted practice in the facility [57]. Furthermore, it is also well recognised that structural and policy factors such as social trends, the extent of baby food marketing, and policies influencing health care or employment environments are important determinants of breastfeeding practices; it is not only the attitudes and decisions of individual mothers and their families [51].

A key contribution of this study is that it presents data and analyses which help draw links from comprehensively measured promotional activities demonstrated in Thailand [8] across to Thai mothers’ opinions and formula feeding practices. It is one of few studies in which data were collected on mothers’ exposure to marketing and their opinions on marketing, as well as on their infant feeding practices. Unlike many studies of baby food marketing exposures, a study strength is that it collected data which allows some statistical inferences to be drawn about the effects of marketing, both on both mothers’ attitudes and on their behaviours. Study design and data collections also allowed adjustment for key sociodemographic factors and other variables such as marketing and advice from significant others. A further strength of this study is that it collected comprehensive data on a variety of marketing techniques using a study design protocol for monitoring baby food marketing developed by the WHO, which is one of the most commonly used BMS marketing assessment tools [58]. This means it captures baby food marketing exposures comprehensively and systematically, and is closely comparable with several studies using elements of the same protocol in other countries [59–62].

Limitations of this study are as follows: first, the NetCode toolkit protocol recommendations for periodic study [36] are that it should be conducted in the capital or largest city of the country because baby food marketing in big cities is more common than in small cities. Therefore, this study collected data in Bangkok only. Consequently, baby food marketing practice in other provinces or other regions was excluded. According to Thailand’s Multiple Indicator Cluster Survey 2019, the percentage of infants under six months of age who are exclusively breastfed (received only breastmilk during the previous day), in each region was different: Bangkok = 26.4%; Centre = 8%; North = 16.5%; Northeast = 14.2%; and South = 14.1% [1]. One reason for this was that there may be differences in baby food marketing in other areas of Thailand. Furthermore, the protocol suggests that mothers of children under 24 months old should be sampled from the 33 selected health facilities providing well-child services. It may result in sampling bias if attendance at such facilities varies among relevant population subgroups.

Secondly, mothers were asked about their experiences in baby food marketing in the past six months. Consequently, mothers may not report their actual experiences of marketing or their infant feeding practices correctly because they might be unable to remember some details, or they might remember incorrectly. Furthermore, the interviews were held in the waiting room of the vaccination clinics which is a public space. Although our questions did not involve collecting confidential information such as their name or contact information, some mothers might hesitate to answer about feeding history or BMS marketing experience in the public area. Therefore, there is the possibility of social desirability bias in responses about marketing, or infant feeding practices.

Importantly, as a cross-sectional study, a limitation is that our results can only report associations between marketing and infant feeding practices and cannot prove causation. Likewise, there may be reverse causality in some measures, in that mothers seeking infant feeding advice, or recalling their exposure to marketing, may have been experiencing more difficulties with breastfeeding, have less confidence in infant care and feeding, and / or found marketing more salient. Even so, our study showed strong associations between some types of marketing and opinions or feeding practices, adding to a growing literature with consistently similar findings from other countries and study types. Evidence from multiple other studies on the specific links between CMF marketing in health settings and mothers’ infant and young child feeding practices further strengthens the argument that Thai mothers’ exposure to CMF marketing activity in maternity care facilities influences them to use these products. Theories of how mothers’ opinions on infant feeding are formed and interact with practical realities such as education or income also provide plausible explanations for our findings on social determinants of higher CMF use, such as by lesser educated, middle-income, or employed mothers. Future research needs to extend the study area to other provinces in all regions of Thailand, including small provinces. More detailed research focusing on mothers’ perceptions and experiences of baby food marketing in health facilities and through the media in Thailand would also yield a better understanding of how marketing influences the attitudes and behaviours on infant and young child feeding, as these are important sources of behaviour change. Investigating employed mothers’ experience of breastfeeding, family support, and marketing in Thailand would also be worthwhile because they are more pressed for time and hence more likely to be vulnerable and influenced by the marketing of commercial baby foods. Paid lactation breaks and maternity leave are also key factors affecting breastfeeding women that warrant further investigation in Thailand.

## Conclusions and policy implications

This study examined the links between baby food marketing and infant feeding practices in Bangkok, Thailand, using a cross-sectional study of mothers of infants and young children in Bangkok, Thailand. The main findings are that marketing exposure appeared to strongly influence mother’s opinions about commercial baby food products and their feeding decisions and behaviours. Advice about formula from others, particularly, health professional is very important in leading mothers to have positive attitudes toward formula. Marketing at health facilities, and being in formal employment, were most strongly related to a higher likelihood of mothers feeding formula after adjusting for sociodemographic characteristics and other variables affecting feeding decisions.

An increased extent and global pervasiveness of CMF marketing, including in Thailand, is occurring at the same time as a global boom in CMF sales [63]. The causal effects of marketing on product use are well known for some other commercial products, such as tobacco. Together with other research [64, 65], our findings of associations between highly prevalent CMF marketing and mothers’ CMF feeding opinions and behaviours in Thailand suggest that inappropriate marketing is influencing the increased use of these products, and reducing breastfeeding.

Our results suggest prioritising an urgent need to address the problem of marketing in health facilities particularly such as through health professional education and training and through facility policies of BFHI and Ten Steps implementation.

There are also important other health policy implications from this study. One is that the Ministry of Public Health could much more actively monitor and enforce the Act, and digital-marketing monitoring might be strengthened. Of particular importance is that the Ministry of Public Health should strengthen the implementation of relevant existing measures such as the Baby-Friendly Hospital Initiative for ensuring that health facilities will not be employed to promote baby food marketing; as well, maternity leave should be extended to at least six months to ensure that each mother will be able to live with and feed breastmilk to their child longer. Also, mothers, families, and health professionals should be educated comprehensively about the importance of breastfeeding, with wider implementation of health professional training on the Code, and the Act. Moreover, government organisations and relevant Thai officials might learn or exchange experiences with other countries or international organisations such as Alive and Thrive about training personnel on how to monitor compliance with the Code and the Act.

More broadly this study also suggests the importance of ensuring adequate maternity protection is available to all women in Thailand including nursing breaks.

## Supplementary Information

**Additional file 1: Table S1.** Opinion of mothers on formula, by characteristics of mother and type of marketing experienced. **Table S2**. Feeding behaviours. **Table S3**. Mothers’ characteristics and giving formula.

## Data Availability

Not applicable.

## Abbreviations

BFHI: Baby-Friendly Hospital Initiative
BMS: Breastmilk substitutes
CCF: Commercial complementary food
The Code: The International Code of Marketing of Breast-Milk Substitutes and subsequent World Health Assembly resolutions
CMF: Commercial milk formula
CRC: Committee on the Rights of the Child
GUM: Growing-up milks
NetCode: The Network for Global Monitoring and Support for Implementation of the International Code of Marketing of Breast-milk Substitutes and subsequent relevant World Health Assembly Resolutions
RSP: Retail Sale Price
THB: Thai Baht
Thailand MICS: Thailand Multiple Indicator Cluster Survey
WHA: World Health Assembly
WHO: World Health Organization
UNICEF: United Nations International Children’s Emergency Fund
USD: United States dollar
