# Supplementary material for: Effect of baby food marketing exposure on infant and young child feeding regimes in Bangkok, Thailand
Source: Int Breastfeed J. 2022 Sep 1;17:64. doi: 10.1186/s13006-022-00503-7 (PMC9435428; doi:10.1186/s13006-022-00503-7)
Supplement: Supplementary file 1 — Additional file 1: Table S1. Opinion of mothers on formula, by characteristics of mother and type of marketing experienced. Table S2. Feeding behaviours. Table S3. Mothers’ characteristics and giving formula. [file 13006_2022_503_MOESM1_ESM.docx]

**Additional tables**

[Table S1 Opinion of mothers on formula, by characteristics of mother and type of marketing experienced 1](#_Toc99915176)

[Table S2 Feeding behaviours 4](#_Toc99915177)

[Table S3 Mothers’ characteristics and giving formula. 6](#_Toc99915178)

Table S1 Opinion of mothers on formula, by characteristics of mother and type of marketing experienced

|  | Opinion of mothers on formula | | | |
| --- | --- | --- | --- | --- |
|  | Unfavorable | | Favorable | |
|  | N | % | N | % |
| ***Characteristics of mother and opinion on formula*** | | | | |
| **Mothers’ age** |  |  |  |  |
| <20 years | 14 | 45.2 | 17 | 54.8 |
| 20-29 years | 77 | 43.0 | 102 | 57.0 |
| 30 years and above | 53 | 44.2 | 67 | 55.8 |
| **Education level** |  |  |  |  |
| Primary school or lower | 24 | 43.6 | 31 | 56.4 |
| Secondary school or diploma | 94 | 42.0 | 130 | 58.0 |
| Bachelor degree or higher | 25 | 52.1 | 23 | 47.9 |
| **Marital status** |  |  |  |  |
| live without couple | 13 | 43.3 | 17 | 56.7 |
| live as a couple | 131 | 43.7 | 169 | 56.3 |
| **Total number of household members** |  |  |  |  |
| 1-5 persons | 89 | 39.7 | 135 | 60.3 |
| 6 persons and above | 55 | 51.9 | 51 | 48.1 |
| **Type of household** |  |  |  |  |
| nuclear family | 69 | 42.1 | 95 | 57.9 |
| extended family | 75 | 45.5 | 90 | 54.5 |
| **CURRENT occupation/employment status of mothers** |  |  |  |  |
| Non-employed/ student | 73 | 43.2 | 96 | 56.8 |
| Formal work | 39 | 43.8 | 50 | 56.2 |
| Informal work | 30 | 43.5 | 39 | 56.5 |
| **Household monthly incomes** |  |  |  |  |
| 0 – 15,000 THB | 48 | 50.5 | 47 | 49.5 |
| 15,001 – 50,000 THB | 66 | 34.7 | 124 | 65.3 |
| More than 50,000 THB | 30 | 66.7 | 15 | 33.3 |
| **Types of a hospital where children were born** |  |  |  |  |
| Public hospitals/clinics | 121 | 43.5 | 157 | 56.5 |
| Private hospitals/clinics | 22 | 45.8 | 26 | 54.2 |
| ***Experience of advice and marketing, and opinion on formula*** | | | | |
| **Advice about formula from others** |  |  |  |  |
| No experience | 119 | 46.7 | 136 | 53.3 |
| Having experience | 24 | 32.4 | 50 | 67.6 |
| **Advice about commercially-prepared complementary food from others** |  |  |  |  |
| No experience | 115 | 43.9 | 147 | 56.1 |
| Having experience | 29 | 42.6 | 39 | 57.4 |
| **Marketing from health facilities** |  |  |  |  |
| No experience | 107 | 42.5 | 145 | 57.5 |
| Having experience | 36 | 48.6 | 38 | 51.4 |
| **Marketing from media** |  |  |  |  |
| No experience | 24 | 41.4 | 34 | 58.6 |
| Having experience | 116 | 43.5 | 151 | 56.5 |
| **Companies' social group and events** |  |  |  |  |
| No experience | 38 | 47.5 | 42 | 52.5 |
| Having experience | 27 | 45.0 | 33 | 55.0 |
| **Free baby food sample** |  |  |  |  |
| No experience | 108 | 44.3 | 136 | 55.7 |
| Having experience | 36 | 41.9 | 50 | 58.1 |
| **Free coupon relating to baby food products or companies** |  |  |  |  |
| No experience | 134 | 43.9 | 171 | 56.1 |
| Having experience | 9 | 37.5 | 15 | 62.5 |
| **Free gift relating to baby food products or companies** |  |  |  |  |
| No experience | 127 | 43.9 | 162 | 56.1 |
| Having experience | 15 | 38.5 | 24 | 61.5 |

Table S2 Feeding behaviours

| **Feeding history** | **N (%)** |
| --- | --- |
| **Your child has ever been breastfed (N= 330)** |  |
| Yes | 321 (97.3) |
| No | 9 (2.7) |
| **Your child is still being breastfed (N= 321)** |  |
| Yes | 159 (49.5) |
| No | 162 (50.5) |
| **Your child STOPPED breastfeeding at (N= 162)** |  |
| 0-1 month | 33 (20.4) |
| 2-3 months | 71 (43.8) |
| 4-5 months | 14 (8.6) |
| 6-11 months | 37 (22.8) |
| 12 months and above | 7 (4.4) |
| **You give your child any formula (N= 330)** |  |
| Yes | 246 (74.5) |
| No | 84 (25.5) |
| **Your child STARTED formula at (N= 246)** |  |
| 0-1 month | 73 (29.7) |
| 2-3 months | 81 (32.9) |
| 4-5 months | 34 (13.8) |
| 6 months and above | 58 (23.6) |
| **Frequency of formula feeding (N= 246)** |  |
| Every feed | 142 (57.7) |
| Most feeds | 36 (14.6) |
| Occasional feeds | 68 (27.7) |
| **You give your child any home-prepared complementary/baby food (N= 330)** |  |
| Yes | 225 (68.2) |
| No | 105 (31.8) |
| **Your child STARTED home-prepared complementary/baby food at (N=225)** |  |
| 0-1 month | 3 (1.3) |
| 2-3 months | 25 (11.1) |
| 4-5 months | 43 (19.1) |
| 6 months and above | 154 (68.5) |
| **Frequency of home-prepared complementary feeding (N= 225)** |  |
| Every feed | 59 (26.2) |
| Most feeds | 57 (25.3) |
| Occasional feeds | 109 (48.5) |
| **You give your child any commercial-prepared complementary/baby food (N= 330)** |  |
| Yes | 130 (39.4) |
| No | 200 (60.6) |
| **Your child STARTED commercial-prepared complementary food at** **(N= 130)** |  |
| 0-1 month | 1 (0.8) |
| 2-3 months | 12 (9.2) |
| 4-5 months | 33 (25.4) |
| 6 months and above | 84 (64.6) |
| Total | 130 (100) |
| **Frequency of commercial-prepared complementary feeding (N= 130)** |  |
| Every feed | 9 (6.9) |
| Most feeds | 20 (15.4) |
| Occasional feeds | 101 (77.7) |

Table S3 Mothers’ characteristics and giving formula.

| **Mothers’ characteristics** | **Giving formula** | | | |
| --- | --- | --- | --- | --- |
|  | **No** | | **Yes** | |
|  | **N** | **%** | **N** | **%** |
| ***Characteristics of mother and opinion on formula*** | | | | |
| **Mothers’ ages** |  |  |  |  |
| <20 years | 6 | 19.4 | 25 | 80.6 |
| 20-29 years | 45 | 25.1 | 134 | 74.9 |
| 30 years and above | 33 | 27.5 | 87 | 72.5 |
| **Education level** |  |  |  |  |
| Primary school or lower | 17 | 30.9 | 38 | 69.1 |
| Secondary school or diploma | 56 | 25.0 | 168 | 75.0 |
| Bachelor degree or higher | 9 | 18.8 | 39 | 81.2 |
| **Marital status** |  |  |  |  |
| live without couple | 5 | 16.7 | 25 | 83.3 |
| live as a couple | 79 | 26.3 | 221 | 73.7 |
| **Total number of household members** |  |  |  |  |
| 1-5 persons | 56 | 25.0 | 168 | 75.0 |
| 6 persons and above | 28 | 26.4 | 78 | 73.6 |
| **Type of household** |  |  |  |  |
| nuclear family | 45 | 27.4 | 119 | 72.6 |
| extended family | 38 | 23.0 | 127 | 77.0 |
| **CURRENT occupation/employment status of mothers** |  |  |  |  |
| Non-employed/ student | 51 | 30.2 | 118 | 69.8 |
| Formal work | 11 | 12.4 | 78 | 87.6 |
| Informal work | 21 | 30.4 | 48 | 69.6 |
| **Household monthly incomes** |  |  |  |  |
| 0 – 15,000 THB | 32 | 33.7 | 63 | 66.3 |
| 15,001 – 50,000 THB | 43 | 22.6 | 147 | 77.4 |
| More than 50,000 THB | 9 | 20.0 | 36 | 80.0 |
| **Types of a hospital where children were born** |  |  |  |  |
| Public hospitals/clinics | 75 | 27.0 | 203 | 73.0 |
| Private hospitals/clinics | 6 | 12.5 | 42 | 87.5 |
| ***Experience of advice and marketing, and opinion on formula*** | | | | |
| **Advice about formula from other people** |  |  |  |  |
| No experience | 64 | 25.1 | 191 | 74.9 |
| Having experience | 20 | 27.0 | 54 | 73.0 |
| **Advice about commercially-prepared complementary food from other people** |  |  |  |  |
| No experience | 73 | 27.9 | 189 | 72.1 |
| Having experience | 11 | 16.2 | 57 | 83.8 |
| **Marketing from health facilities** |  |  |  |  |
| No experience | 68 | 27.0 | 184 | 73.0 |
| Having experience | 15 | 20.3 | 59 | 79.7 |
| **Marketing from media** |  |  |  |  |
| No experience | 24 | 41.4 | 34 | 58.6 |
| Having experience | 57 | 21.4 | 210 | 78.6 |
| **Companies' social group and events** |  |  |  |  |
| No experience | 14 | 17.5 | 66 | 82.5 |
| Having experience | 15 | 25.0 | 45 | 75.0 |
| **Free baby food sample** |  |  |  |  |
| No experience | 65 | 26.6 | 179 | 73.4 |
| Having experience | 19 | 22.1 | 67 | 77.9 |
| **Free coupon relating to baby food products or companies** |  |  |  |  |
| No experience | 80 | 26.2 | 225 | 73.8 |
| Having experience | 3 | 12.5 | 21 | 87.5 |
| **Free gift relating to baby food products or companies** |  |  |  |  |
| No experience | 72 | 24.9 | 217 | 75.1 |
| Having experience | 11 | 28.2 | 28 | 71.8 |
